# Supplementary material for: The characteristics and extent of food industry involvement in peer-reviewed research articles from 10 leading nutrition-related journals in 2018
Source: PLoS One. 2020 Dec 16;15(12):e0243144. doi: 10.1371/journal.pone.0243144 (PMC7743938; doi:10.1371/journal.pone.0243144)
Supplement: S1 Table — (DOCX) [file pone.0243144.s001.docx]

**S1 Table.** Definitions of categories used to classify organisations from the food industry

| **Food industry sector** | **Definition** |
| --- | --- |
| Dairy | This includes organisations that are involved the production, processing, and promotion of dairy products. |
| Dietary supplement manufacturing | This includes organisations that are involved in the production, processing and promotion of infant formula, vitamin and mineral supplements, probiotics, and protein shakes/bars. |
| Food chemical suppliers and food technology companies | This includes organisations that are involved in the production, processing and promotion of products related to novel and functional food ingredients. |
| Food retail | This includes all supermarkets, grocery stores, restaurants, fast food outlets, and catering companies. |
| Meat and livestock | This includes organisations that are involved in the production, processing, and promotion of animal products and meat. |
| Non-alcoholic beverage manufacturing | This includes organisations involved in processing, packaging, and distributing non-alcoholic beverages. |
| Primary production (non-dairy, non-meat) | This includes organisations that are involved in the production, processing and promotion of raw food materials including, but not limited to, eggs, potatoes, oil, nuts, avocado, and maize. |
| Processed food manufacturing | This includes organisations involved in processing, packaging, and distributing processed packaged foods. |
| Other | Any food industry company or organisation that did not fit within the above categories. |
