# Supplementary material for: The characteristics and extent of food industry involvement in peer-reviewed research articles from 10 leading nutrition-related journals in 2018
Source: PLoS One. 2020 Dec 16;15(12):e0243144. doi: 10.1371/journal.pone.0243144 (PMC7743938; doi:10.1371/journal.pone.0243144)
Supplement: S2 Table — (DOCX) [file pone.0243144.s002.docx]

**S2 Table.** Food industry actors identified as involved in research studies in the top 10 most-cited nutrition- and dietetics-related journals in 2018, by food industry sector and actor classification

| **Food industry sector** | **Organisation ^1^** | **Classification ^2^** |
| --- | --- | --- |
| **Dairy** | Alberta Milk | Trade/Industry Association |
|  | Arla Foods | Large corporation |
|  | Dairy Australia | Trade/Industry Association |
|  | Dairy Farmers of Canada | Trade/Industry Association |
|  | Dairy Farmers of Ontario | Trade/Industry Association |
|  | Dairy Research Cluster Initiative | Trade/Industry Association |
|  | Dairy Research Institute | Trade/Industry Association |
|  | Danish Dairy Research Council | Trade/Industry Association |
|  | Danone | Large corporation |
|  | Fonterra | Large corporation |
|  | Friesland Campina | Small corporation/other entity |
|  | Leprino Foods | Small corporation/other entity |
|  | National Interprofessional Centre of the Dairy Industry | Trade/Industry Association |
|  | The West Azarbaijan Dairy Company | Small corporation/other entity |
|  | The Yoghurt Council | Trade/Industry Association |
|  | UK Dairy Council | Trade/Industry Association |
|  | Ultima Foods | Small corporation/other entity |
|  | US Dairy Export Council | Trade/Industry Association |
|  | US National Dairy Council | Trade/Industry Association |
|  | Valio Oy | Large corporation |
|  | World Dairy Platform | Trade/Industry Association |
|  | Yoplait | Small corporation/other entity |
| **Dietary supplement manufacturing** | Abbott | Large corporation |
|  | Atrium Innovations | Small corporation/other entity |
|  | Beneo | Large corporation |
|  | BioActor | Small corporation/other entity |
|  | Cambrooke Therapeutics | Small corporation/other entity |
|  | DuPoint Nutrition & Biosciences | Large corporation |
|  | General Nutrition Coporation | Small corporation/other entity |
|  | Herbalife | Large corporation |
|  | InovoBiologic | Small corporation/other entity |
|  | International Alliance of Dietary Supplement Association | Trade/Industry Association |
|  | Jamieson Wellness | Small corporation/other entity |
|  | Mead Johnson Nutritionals | Small corporation/other entity |
|  | Metabolic Technologies Inc | Small corporation/other entity |
|  | MS Prebiotics | Small corporation/other entity |
|  | MYOS Corporation | Small corporation/other entity |
|  | Natural Factors | Small corporation/other entity |
|  | Nestlé-Novartis | Large corporation |
|  | Nordic Naturals | Small corporation/other entity |
|  | Nutricia (Danone) | Large corporation |
|  | NutriSci | Small corporation/other entity |
|  | Oy Verman Ab | Small corporation/other entity |
|  | Prolacta Bioscience | Small corporation/other entity |
|  | The Juice Plus+ Company | Small corporation/other entity |
|  | Vitaflo UK | Small corporation/other entity |
|  | Winclove & Gut Flora Foundation | Small corporation/other entity |
| **Food chemical suppliers and food technology companies** | Food for Health Ireland | Small corporation/other entity |
|  | GoodMills Innovation | Small corporation/other entity |
|  | Holistic Bio | Small corporation/other entity |
|  | Ingredion Incorporated | Large corporation |
| **Food retail** | Eroski Distribution | Small corporation/other entity |
|  | Institute of Grocery Distribution | Small corporation/other entity |
|  | Loblaw Companies | Large corporation |
|  | Provigo-Loblaws (Loblaw Companies) | Large corporation |
|  | Trade Association of Dutch Catering Organisations | Trade/Industry Association |
|  | Agropur Dairy Cooperative | Trade/Industry Association |
| **Meat and livestock** | Alberta Livestock and Meat Agency | Trade/Industry Association |
|  | Beef Checkoff | Trade/Industry Association |
|  | Meat & Livestock Australia | Trade/Industry Association |
|  | National Cattlemen’s Beef Association | Trade/Industry Association |
|  | National Pork Board | Trade/Industry Association |
|  | North Dakota Beef Commission | Trade/Industry Association |
|  | Pork Checkoff | Trade/Industry Association |
|  | Primo | Large corporation |
| **Non-alcoholic beverage manufacturing** | American Beverage Association | Trade/Industry Association |
|  | Coca-Cola | Large corporation |
|  | PepsiCo | Large corporation |
| **Primary production (non-dairy, non-meat)** | Alliance for Potato Research Education | Trade/Industry Association |
|  | Almond Board of Australia | Trade/Industry Association |
|  | Almond Board of California | Trade/Industry Association |
|  | Alphonsa Cashew Industries | Trade/Industry Association |
|  | American Egg Board & Egg Nutrition Center | Trade/Industry Association |
|  | American Peanut Council | Trade/Industry Association |
|  | Australian Egg Corporation | Trade/Industry Association |
|  | Borges | Small corporation/other entity |
|  | British Egg Information Service | Trade/Industry Association |
|  | California Strawberry Commission | Trade/Industry Association |
|  | California Walnut Commission | Trade/Industry Association |
|  | Canola Council of Canada | Trade/Industry Association |
|  | Cashew Export Promotion Council of India | Trade/Industry Association |
|  | Cashew Manufacturers Association | Trade/Industry Association |
|  | Cobram Estate | Small corporation/other entity |
|  | Crisolar Spain | Small corporation/other entity |
|  | Egg Nutrition Council | Trade/Industry Association |
|  | Flax Council of Canada | Trade/Industry Association |
|  | Hass Avocado Board | Trade/Industry Association |
|  | Hazelnut Marketing Board of Oregon | Trade/Industry Association |
|  | International Maize and Wheat Improvement Center | Trade/Industry Association |
|  | International Nut & Dried Fruit Council | Trade/Industry Association |
|  | International Tree Nut Council Nutrition Research and Education Foundation | Trade/Industry Association |
|  | Kerala Nut Food Company | Small corporation/other entity |
|  | Morella Nuts | Small corporation/other entity |
|  | Netherlands Sugar Foundation | Trade/Industry Association |
|  | Nut and Dried Fruit Foundation | Trade/Industry Association |
|  | Nuts for Life | Trade/Industry Association |
|  | Patrimonio Communal Olivarero | Trade/Industry Association |
|  | Pistachio growers | Trade/Industry Association |
|  | Potatoes USA | Trade/Industry Association |
|  | Pristine Gourmet | Small corporation/other entity |
|  | Pulse Canada | Trade/Industry Association |
|  | Saskatchewan Pulse Growers | Trade/Industry Association |
|  | Solae | Small corporation/other entity |
|  | Soy Foods Association of North America | Trade/Industry Association |
|  | Sugar Association | Trade/Industry Association |
|  | The Ginger Network | Trade/Industry Association |
|  | The Grain Food Foundation | Trade/Industry Association |
|  | The Peanut Institute | Trade/Industry Association |
|  | Welsh, Holme & Clark Co | Small corporation/other entity |
|  | Western India Cashew Company & Intersnack Procurement | Small corporation/other entity |
|  | Zespri International | Large corporation |
| **Processed food manufacturing** | ACH Food Companies | Small corporation/other entity |
|  | Ajinomoto | Large corporation |
|  | Atkins | Small corporation/other entity |
|  | Barilla | Large corporation |
|  | Bledina | Small corporation/other entity |
|  | Bunge Limited | Large corporation |
|  | Campbell Soup Co | Large corporation |
|  | Carbery Group Ltd. | Small corporation/other entity |
|  | Cereal Partners Worldwide | Large corporation |
|  | Clif Bar Company | Small corporation/other entity |
|  | Conagra Brands | Large corporation |
|  | Fazer | Small corporation/other entity |
|  | Ferrero | Large corporation |
|  | Fresystem SpA | Small corporation/other entity |
|  | General Mills | Large corporation |
|  | Gerber | Small corporation/other entity |
|  | Glanbia Plc | Large corporation |
|  | Grupo Nutresa | Large corporation |
|  | Haine Celestial Group | Large corporation |
|  | Kellogg’s | Large corporation |
|  | Kerry Group | Large corporation |
|  | Mars | Large corporation |
|  | McCormick Spice | Large corporation |
|  | Nestec (part of Nestlé) | Large corporation |
|  | Nestlé | Large corporation |
|  | Nutrisystem | Small corporation/other entity |
|  | Nutrition et Sante | Small corporation/other entity |
|  | Quaker (PepsiCo) | Large corporation |
|  | Sanitarium Company | Small corporation/other entity |
|  | Simplot Australia | Large corporation |
|  | The Dutch Bakery Center | Trade/Industry Association |
|  | Twodads | Small corporation/other entity |
|  | Unilever | Large corporation |
|  | Whitewave Foods | Large corporation |
| **Other** | Advanced Foods and Materials Network | Small corporation/other entity |
|  | Amway | Large corporation |
|  | British Nutrition Foundation | Trade/Industry Association |
|  | DSM Food Specialities | Small corporation/other entity |
|  | DSM Nutritional products | Small corporation/other entity |
|  | European Stevia Association | Trade/Industry Association |
|  | Global Stevia Research Institute | Trade/Industry Association |
|  | HarvestPlus | Small corporation/other entity |
|  | International Life Sciences Institute | Trade/Industry Association |
|  | International Plant Sterol and Stanol Association | Trade/Industry Association |
|  | International Sweeteners Association | Trade/Industry Association |
|  | Knowledge Centre Sugar & Nutrition | Small corporation/other entity |
|  | Nutrition Impact | Small corporation/other entity |
|  | Omega Protein Corporation | Small corporation/other entity |
|  | PureCircle | Small corporation/other entity |
|  | Sugar Nutrition UK | Trade/Industry Association |
|  | Top Institute Food and Nutrition | Trade/Industry Association |
|  | US Whey Protein Research Consortium | Trade/Industry Association |

^1^ Categorisation based on the relevant industry sector for the majority of the products / services of an organisation or the organisations that they represent.

^2^ Large corporation = annual global revenue estimated at >USD1 billion; Small corporation/other entity = annual global revenue estimated at < USD1 billion.
