# Supplementary material for: The characteristics and extent of food industry involvement in peer-reviewed research articles from 10 leading nutrition-related journals in 2018
Source: PLoS One. 2020 Dec 16;15(12):e0243144. doi: 10.1371/journal.pone.0243144 (PMC7743938; doi:10.1371/journal.pone.0243144)
Supplement: S3 Table — (DOCX) [file pone.0243144.s003.docx]

**S3 Table.** The top 10 most-cited nutrition- and dietetics-related journals in 2018 and their declared involvement with the food industry

| **Journal** | **% of articles with food industry involvement ^1^** | **Statement regarding potential editorial conflicts of interest on journal website** | **Editors with declared involvement with food industry ^2^** | **Other journal links to food industry** |
| --- | --- | --- | --- | --- |
| *The Journal of Nutrition* | 28.3% | Yes | Yes | Published by The *American Society for Nutrition* which includes multiple food industry sponsors |
| *Nutrition Reviews* | 24.5% | No | Yes | Published on behalf of the [*International Life Sciences Institute*](https://en.wikipedia.org/wiki/International_Life_Sciences_Institute) – an organisation with strong links to the food industry |
| *The American Journal of Clinical Nutrition* | 16.7% | Yes | Yes | Published by The *American Society for Nutrition* which includes multiple food industry sponsors |
| *Clinical Nutrition* | 16.5% | No | No |  |
| *Obesity* | 9.5% | Yes | Yes |  |
| *Advances in Nutrition* | 9.4% | Yes | Yes | Published by The *American Society for Nutrition* which includes multiple food industry sponsors |
| *Nutrition Research Reviews* | 4.8% | No | No |  |
| *International Journal of Obesity* | 4.9% | No | Yes |  |
| *International Journal of Behavioral Nutrition and Physical Activity* | 4.0% | No | No |  |
| *Paediatric Obesity* | 3.8% | Yes (Editor-in-Chief only) | No |  |

^1^ In relation to original research articles published in 2018.

^2^ Involvement with food industry based on biographical information provided on the journal website and/or on the website of each editor’s primary affiliation.
