# Supplementary material for: The characteristics and extent of food industry involvement in peer-reviewed research articles from 10 leading nutrition-related journals in 2018
Source: PLoS One. 2020 Dec 16;15(12):e0243144. doi: 10.1371/journal.pone.0243144 (PMC7743938; doi:10.1371/journal.pone.0243144)
Supplement: S4 Table — (DOCX) [file pone.0243144.s004.docx]

**S4 Table.** Food industry actors identified as being involved in the top 10 most-cited nutrition- and dietetics-related journals in 2018

| **Classification ^1^** | **Organisations identified (n, % of total)** | **Number of times food industry actors were identified as having involvement**  **(n, % of total) ^2^** |
| --- | --- | --- |
| Large corporations | 37, 23.0% | 197, 47.8% |
| Trade/industry associations | 67, 41.6% | 50, 36.4% |
| Small corporations/other entity | 57, 35.4% | 65, 15.8% |
| **Total** | **161, 100%** | **412, 100%** |

^1^ Large corporation = annual global revenue estimated at >USD1 billion; Small corporation/other entity = annual global revenue estimated at < USD1 billion.

^2^ In many cases, multiple food industry actors were involved in a single article. Refer to **S5 Table** for further details of involvement of individual food industry actors.
