# Supplementary material for: The characteristics and extent of food industry involvement in peer-reviewed research articles from 10 leading nutrition-related journals in 2018
Source: PLoS One. 2020 Dec 16;15(12):e0243144. doi: 10.1371/journal.pone.0243144 (PMC7743938; doi:10.1371/journal.pone.0243144)
Supplement: S5 Table — (DOCX) [file pone.0243144.s005.docx]

**S5 Table.** Food industry actors identified as being involved in more than 1% of articles examined in the top 10 most-cited nutrition- and dietetics-related journals in 2018

| **Organisation** | **Industry sector** | **Classification ^4^** | **Number of articles in which involvement was identified** | **Percentage** |
| --- | --- | --- | --- | --- |
| Nestlé ^1^ | Processed food manufacturing | Large corporation | 44 | 22.5% |
| Danone ^2^ | Dairy / dietary supplement manufacturing | Large corporation | 43 | 21.9% |
| Abbott | Dietary supplement manufacturing | Large corporation | 23 | 11.7% |
| PepsiCo ^3^ | Non-alcoholic beverage manufacturing/ Processed food manufacturing | Large corporation | 16 | 8.2% |
| US National Dairy Council | Dairy | Trade/Industry Association | 13 | 6.6% |
| American Egg Board/Egg Nutrition Center | Primary production (non-dairy, non-meat) | Trade/Industry Association | 13 | 6.6% |
| Unilever | Processed food manufacturing | Large corporation | 12 | 6.1% |
| Coca-Cola | Non-alcoholic beverage manufacturing | Large corporation | 9 | 4.6% |
| Dairy Farmers of Canada | Dairy | Trade/Industry Association | 9 | 4.6% |
| California Walnut Commission | Primary production (non-dairy, non-meat) | Trade/Industry Association | 8 | 4.1% |
| National Cattlemen's Beef Association | Meat and livestock | Trade/Industry Association | 7 | 3.6% |
| Beef Checkoff | Meat and livestock | Trade/Industry Association | 7 | 3.6% |
| Kellogg's | Processed food manufacturing | Large corporation | 6 | 3.1% |
| Arla | Dairy | Large corporation | 5 | 2.6% |
| Canola Council of Canada | Primary production (non-dairy, non-meat) | Trade/Industry Association | 5 | 2.6% |
| Flax Council of Canada | Primary production (non-dairy, non-meat) | Trade/Industry Association | 5 | 2.6% |
| Conagra Brands | Processed food manufacturing | Large corporation | 5 | 2.6% |
| Dairy Australia | Dairy | Trade/Industry Association | 5 | 2.6% |
| Hass Avocado Board | Primary production (non-dairy, non-meat) | Trade/Industry Association | 4 | 2.0% |
| Friesland | Dairy | Small corporation/other entity | 4 | 2.0% |
| International Sweeteners Association | Other | Trade/Industry Association | 4 | 2.0% |
| DSM Nutritional Products | Other | Small corporation/other entity | 4 | 2.0% |
| Barilla | Processed food manufacturing | Large corporation | 4 | 2.0% |
| Almond board of California | Primary production (non-dairy, non-meat) | Trade/Industry Association | 4 | 2.0% |
| International Life Sciences Institute | Other | Trade/Industry Association | 3 | 1.5% |
| Top Institute Food and Nutrition | Other | Trade/Industry Association | 3 | 1.5% |
| National Pork Board | Meat & livestock | Trade/Industry Association | 3 | 1.5% |
| McCormick Spice | Processed food manufacturing | Large corporation | 3 | 1.5% |
| International Nut & Dried Fruit Council | Primary production (non-dairy, non-meat) | Trade/Industry Association | 3 | 1.5% |
| Leprino Foods | Dairy | Small corporation/other entity | 3 | 1.5% |
| Pulse Canada | Primary production (non-dairy, non-meat) | Trade/Industry Association | 3 | 1.5% |
| American Beverage Association | Non-alcoholic beverage manufacturing | Trade/Industry Association | 2 | 1.0% |
| Fonterra | Dairy | Large corporation | 2 | 1.0% |

^1^ Includes Nestlé and Nestec.

^2^ Includes Danone, Nutritica and Danone-Nutricia.

^3^ Includes PepsiCo and Quaker.

^4^ Large corporation = annual global revenue estimated at >USD1 billion; Small corporation/other entity = annual global revenue estimated at < USD1 billion.
