# Supplementary material for: The characteristics and extent of food industry involvement in peer-reviewed research articles from 10 leading nutrition-related journals in 2018
Source: PLoS One. 2020 Dec 16;15(12):e0243144. doi: 10.1371/journal.pone.0243144 (PMC7743938; doi:10.1371/journal.pone.0243144)
Supplement: S6 Table — (DOCX) [file pone.0243144.s006.docx]

**S6 Table.** Primary topic area of the random sample of articles without food industry involvement^1^

| Primary topic area of article | Articles with findings **favourable** to the food industry | Articles with findings **unfavourable** to the food industry | Articles with **mixed** findings with respect to the food industry | Articles with **neutral** findings with respect to the food industry | Articles with findings **not applicable** to food industry interests | **Total** |
| --- | --- | --- | --- | --- | --- | --- |
|  | (n, % of total) | | | | | |
| Nutrients and/or food components, including nutrition supplements ^2^ | Calcium: 1  Fats: 1  Flavinoids: 1  Isoflavones: 2  Magnesium: 1  Non-caloric sweeteners: 1  Protein: 4  Vitamins: 4  **Total: 15, 7.6%** | Breastmilk substitutes: 2  Caffeine: 2  Fats: 3  Phytochemicals: 1  Selenium: 1  **Total: 9, 4.6%** | Fats: 1  Vitamins: 3  **Total: 4, 2.0%** | Beta carotene: 1  Calcium: 3  Choline: 1  Fats: 2  Fibre: 1  Folate: 2  Iodine: 1  Iron: 2  Polyphenols: 1  Protein: 1  Vitamins: 6  Zinc: 1  **Total: 22, 11.2%** | 0, 0.0% | 50, 25.5% |
| Foods and food products (including fruit, vegetables, grains) ^3^ | Coffee: 1  Green tea: 1  Lentils: 1  Walnuts: 1  **Total: 4, 2.0%** | 0, 0.0% | Beans: 1, 0.5% | Milk: 2, 1.0% | 0, 0.0% | 7, 3.6% |
| Physiology and metabolism | 0, 0.0% | 0, 0.0% | 0, 0.0% | 0, 0.0% | 52, 26.5% | 52, 26.5% |
| Diet quality, dietary patterns and related epidemiology | 0, 0.0% | 0, 0.0% | 14, 7.1% | 2, 1.0% | 38, 19.4% | 54, 27.6% |
| Lifestyle, behaviour and environmental influences | 0, 0.0% | 3, 1.5% | 1, 0.5% | 2, 1.0% | 21, 10.7% | 27, 13.8% |
| Methods | 0, 0.0% | 0, 0.0% | 0, 0.0% | 0, 0.0% | 6, 3.1% | 6, 3.1% |
| **Total** | **19, 9.7%** | **12, 6.1%** | **20, 10.2%** | **28, 14.3%** | **117, 59.7%** | **196, 100%** |

^1^ A random sample of articles without industry involvement were selected to match the number of articles with food industry involvement for each journal included in the study.

^2^ The specific nutrients and/or food components that were the topic of each article (and the relevant number of articles related to each one) are noted in each cell.

^3^ The specific foods and/or food products that were the topic of the article (and the relevant number of articles related to each one) are noted in each cell.
