# Supplementary material for: The characteristics and extent of food industry involvement in peer-reviewed research articles from 10 leading nutrition-related journals in 2018
Source: PLoS One. 2020 Dec 16;15(12):e0243144. doi: 10.1371/journal.pone.0243144 (PMC7743938; doi:10.1371/journal.pone.0243144)
Supplement: S7 Table — (DOCX) [file pone.0243144.s007.docx]

**S7 Table.** Nature of the findings in articles with and without ^1^ food industry involvement, by journal

| Journal | Articles with findings **favourable** to the food industry | | Articles with findings **unfavourable** to the food industry | | Articles with **mixed** findings with respect to the food industry | | Articles with **neutral** findings with respect to the food industry | | Articles with findings **not applicable** to food industry interests | |
| --- | --- | --- | --- | --- | --- | --- | --- | --- | --- | --- |
|  | (n, % of total with food industry involvement) | | | | | | | | | |
|  | Industry involvement | | Industry involvement | | Industry involvement | | Industry involvement | | Industry involvement | |
|  | **Yes** | **No** | **Yes** | **No** | **Yes** | **No** | **Yes** | **No** | **Yes** | **No** |
| *The American Journal of Clinical Nutrition* | 23, 62.2% | 6, 16.2% | 5, 13.5% | 5, 13.5% | 4, 10.8% | 4, 10.8% | 0, 0.0% | 8, 21.6% | 5, 13.5% | 14, 37.8% |
| *International Journal of Behavioural Nutrition and Physical Activity* | 2, 40.0% | 0, 0.0% | 0, 0.0% | 2, 40.0% | 0, 0.0% | 0, 0.0% | 0, 0.0% | 0, 0.0% | 3, 60.0% | 3, 60.0% |
| *Advances in Nutrition* | 4, 66.7% | 1, 16.7% | 0, 0.0% | 0, 0.0% | 1, 16.7% | 1, 16.7% | 1, 16.7% | 4, 66.7% | 0, 0.0% | 0, 0.0% |
| *International Journal of Obesity* | 1,10.0% | 0, 0.00% | 0, 0.0% | 0, 0.0% | 0, 0.0% | 1, 10.0% | 0, 0.0% | 0, 0.0% | 9, 90.0% | 9, 90.0% |
| *Nutrition Reviews* | 12, 92.3% | 1, 7.7% | 0, 0.0% | 0, 0.0% | 1, 7.7% | 3, 23.1% | 0, 0.0% | 6, 46.2% | 0, 0.0% | 3, 23.1% |
| *Nutrition Research Reviews* | 1, 100.0% | 0, 0.0% | 0, 0.0% | 0, 0.0% | 0, 0.0% | 0, 0.0% | 0, 0.0% | 0, 0.0% | 0, 0.0% | 1, 100.0% |
| *The Journal of Nutrition* | 37, 58.73% | 5, 7.9% | 6, 9.5% | 2, 3.2% | 4, 6.3% | 8, 12.7% | 4, 6.3% | 4, 6.3% | 12, 19.0% | 44, 69.8% |
| *Obesity* | 3, 13.6% | 1, 4.5% | 1, 4.5% | 0, 0.0% | 3, 13.6% | 1, 4.5% | 0, 0.0% | 0, 0.0% | 15, 68.2% | 20, 90.9% |
| *Paediatric Obesity* | 1, 25.0% | 0, 0.0% | 0, 0.0% | 1, 25.0% | 1, 25.0 | 0, 0.0% | 0, 0.0% | 0, 0.0% | 2, 50.0% | 3, 75.0% |
| *Clinical Nutrition* | 25, 71.4% | 5, 14.3% | 1, 2.9% | 2, 5.7% | 5, 14.3 | 2, 5.7% | 0, 0.0% | 6, 17.1% | 4, 11.4% | 20, 57.1% |
| **Total** | **109, 55.6%** | **19, 9.7%** | **13, 6.6%** | **12, 6.1%** | **19, 9.7%** | **20, 10.2%** | **5, 2.6%** | **28, 14.3%** | **50, 25.5%** | **117, 59.7%** |

^1^ A random sample of articles without industry involvement were selected to match the number of articles with food industry involvement for each journal included in the study.
