# Supplementary material for: The characteristics and extent of food industry involvement in peer-reviewed research articles from 10 leading nutrition-related journals in 2018
Source: PLoS One. 2020 Dec 16;15(12):e0243144. doi: 10.1371/journal.pone.0243144 (PMC7743938; doi:10.1371/journal.pone.0243144)
Supplement: Attachment — Submitted filename: PLOS ONE response to reviewers FINAL.docx [file pone.0243144.s008.docx]

| **Reviewer comment** | | **Authors’ response** | **Changes made to text** |
| --- | --- | --- | --- |
| **Academic editor** | | | |
| 1. | The reviewers felt that this paper makes an important contribution. However, there are a few major issues that require attention:  First, the analysis feels incomplete. In providing a survey of the extent/prevalence of industry involvement in nutrition research, I would expect to see calculations of population proportions with confidence intervals, for example. In measuring the relationship between industry involvement and favourable results, the authors should perform statistical tests of these correlations. Sensitivity testing for non-disclosure, and also different types of industry involvement would strengthen the analysis. | Thank you for the suggestion to consider additional statistical analysis. The results section involved two groups of research articles. The first group (n=196) was ALL research articles from the ten included journals in 2018 that were identified as having food industry involvement. As this group is not a sample, the descriptive statistics provided represent actual percentages and frequencies, not estimates, and it is therefore not necessary to calculate confidence intervals.  The second group (n=196) was a randomly selected matched sample of research articles with no food industry involvement. In response to your suggestion, we have now added in 95% confidence intervals for the proportion of articles in each category of favourability (e.g. favourable or unfavourable to food industry interests) for the randomly selected sample. We have added a new paragraph on statistical methods, and the results section now includes the results from this additional analysis. Table 4 now also includes confidence intervals in relation to the sample of articles with no food industry involvement.  We have now included additional analysis based on type of food industry involvement – refer to our response to your comment 5 below.  We have not performed sensitivity testing for non-disclosure, as we did not have sufficient data available to enable such testing. We have now included additional details in the ‘study limitations’ section regarding non-disclosure. | **Lines 200-206**:  **Statistical analysis**  All articles with food industry involvement were identified from each of the ten included journals, with the frequency and percentage in each category of favourability calculated. For the randomly selected matched sample of research articles with no food industry involvement, we calculated 95% confidence intervals (CIs) for the proportion of articles in each category of favourability (e.g., favourable or unfavourable to food industry interests) using Stata 15.0 (StataCorp, College Station, TX, USA).  **Lines 207-282**:  Various updates to results section to include additional analyses.  **Lines 364-369**:  The analysis relied primarily on the self-disclosure of food industry involvement (through declared conflicts of interests, funding acknowledgments, and author affiliations), with different journals having different disclosure requirements. We did not conduct an analysis of the veracity of each journal’s conflict of interest disclosure requirements, but this warrants further exploration. Importantly, undisclosed food industry involvement cannot be captured using the approach we adopted in this study. |
| 2. | In the Introduction, the paper would be strengthened by adding discussion of the complexity of these issues. For example, unlike Big Tobacco, the 'food industry' encompasses a wide range of entities; it would be helpful to provide a lay of the land and point out that many of the negative public health impacts you describe pertain to multinational corporations. I would also suggest considering how the size/scope of entity (corporation vs trade association vs small company) might be incorporated into your coding. Similarly, greater specificity on the public health impacts and the nature of "food industry interests" would make the impetus for and impact of the study clearer. | We have revised the Introduction to be clearer on the nature of the food industry and potential negative public health impact of food industry involvement in research.  We have conducted additional analysis on the size of organisations identified through our analysis. The size of each organisation has been added to Tables S2 and S5, we have added Table S4 summarising the data by size of the organisation, and we have referred to these results in the Results section. | **Lines 54-61**:  A key driver of poor diets globally has been a nutrition transition characterised by increased consumption of ultra-processed packaged foods (2-4). These foods are manufactured, marketed and sold by a diverse selection of companies and organisations, collectively referred to as the ‘food industry’ (5). Importantly, global food systems are now dominated by a relatively small number of large transnational food companies (2,6). The continued generation of profit by these large food companies typically relies on aggressive marketing of their products and brands, as well as political strategies to create regulatory environments that facilitate their market power (7).  **Lines 76-86**:  However, from a public health perspective, several concerns have been identified regarding food industry involvement in research. These include: 1) the creation of increased marketing opportunities for industry products, many of which are harmful to population health (15); 2) the establishment and nurturing of relationships between the food industry and nutrition researchers that serves to increase perceived industry credibility, reduce industry criticism, and encourage increased dependency on the food industry (16, 17); 3) industry influence over research agendas to preferentially focus on topics likely to benefit industry interests, rather than topics of public health importance (18); 4) industry influence on the methods, conclusions and impact of research in ways that are likely to favour industry interests over and above other factors (9, 19-21); and 5) use of research for political purposes (22, 23).  **Lines 152-157**:  In addition, we classified food industry actors into three categories based on the size and nature of their operations. These included large corporations (with annal global revenue > USD1 billion), trade/industry associations, and small corporations/other entities (annual global revenue < USD1 billion). This classification was based on information obtained from the Euromonitor *Passport* database, supplemented by internet searches of the name of the food industry actor where necessary.  **Lines 245-252**:  Of the 161 food industry actors identified as involved in research articles, the highest proportion (41.6%) were classified as trade/industry associations, 35.4% were classified as small corporations/other entities, and 23.0% were classified as large corporations (Table S4). However, these large corporations were the most frequently involved (47.8% of identified instances of food industry involvement), followed by trade/industry associations (36.4% of identified instances of food industry involvement) and small corporations/other entities (15.8% of identified instances of food industry involvement) (Table S4).  **Lines 286-290**:  Food industry involvement spanned a number of industry sectors, with processed food manufacturing, dietary supplement manufacturing and dairy most often represented. The vast majority of industry involvement was from large corporations and trade/industry associations, rather than smaller corporations. |
| 3. | At points, the authors overstate the contribution this kind of analysis can make. Please clarify that the gap your study fills is the extent, nature and impact of food industry involvement in "published" or "peer reviewed" nutrition research. Presumably, a great deal of industrial research is conducted by the food industry. | We have clarified the aims of the study and the contribution of the study to the literature, and noted additional limitations of the study. Please also refer to our responses to several reviewer comments below. | **Lines 97-102**:  This study aimed to contribute to a growing body of empirical analysis related to food industry involvement in peer-reviewed, published scientific research by systematically identifying the extent of food industry involvement in research articles from a large sample of leading nutrition-related journals.  **Lines 348-355**:  To date, this is the first study to systematically examine the extent of involvement of the food industry in peer-reviewed research articles published in the leading nutrition and dietetics journals. Importantly, much peer-reviewed nutrition research is published outside of the selected nutrition and dietetics journals. Moreover, the study was not designed to identify research with food industry involvement that is published in topic areas outside of nutrition and dietetics, outside of peer-reviewed journals, or that is funded or conducted by the industry but remains unpublished. Accordingly, the study represents only a small and selected analysis of the extent of food industry involvement in nutrition research. |
| 4. | Clarification is requested by the reviewers for a number of the coding categories. | We have addressed each of the reviewers’ comments below, including the addition of a new table (Table 1) that clarifies the coding of principal findings, and more detail of how we assessed whether organisations were part of the food industry. | Refer to responses to reviewer comments below. |
| 5. | I would suggest conducting a sensitivity analysis where you separate out industry funding/employment from financial conflicts of interest with industry. The current analysis seems to elide over some important differences in mechanisms for industry influence. I would expect the latter category to also be more common and yet, perhaps the least influential, thus overstating the findings. | Thank you for this suggestion. We have now added in a new Table (Table 4) that includes an analysis of the nature of the findings (favourable, unfavourable, etc) by type of food industry involvement. We have referred to this table in the Results section, and highlighted differences in types of involvement in the Discussion section. We have moved our original Table 4 that presented similar data by journal, to the Online Supplemental Material (Table S6). | Table 4: ‘Nature of the findings in articles with and without ^1^ food industry involvement, by type of food industry involvement’ was added to the manuscript  Table S6**:** ‘Nature of the findings in articles with and without ^1^ food industry involvement, by journal’ was removed from the main manuscript and added to the Online Supplemental Material  **Lines 193-198**:  Results were analysed by type of food industry involvement and by journal. For the purposes of this analysis of ‘type of food industry involvement’, author affiliations with the food industry and direct funding for the study from the food industry were grouped together (as they were considered more direct involvement) and compared to other types of food industry funding (that were considered less direct involvement).  **Lines 207-282**:  Various changes to results section to refer to these new tables.  **Lines 292-295**:  The proportion of articles with findings considered favourable to the food industry was even higher (66.2%) where industry involvement in the study was more direct (author affiliations or direct funding for the study).  **Lines 404-407**:  Based on the findings of this study, all articles that include any type of food industry involvement warrant close scrutiny from journals, with a particular focus on more direct types of involvement (e.g. author affiliations and direct funding for a study). |
| **Reviewer 1** | | | |
| Overall this is a useful and potentially important paper and should be published. I think it could be strengthened in a few ways. First, I think it is vital that clear definitions are given of “industry affiliated” and whether and how that was investigated. Second, I think the authors might be able to add a few further analyses that would strengthen the paper. I should note, however, that I don’t think these are essential. Finally, I think the tables could be improved to make the presentation simpler and clearer. I would also suggest greater attention be given to the variation in levels of favourable industry research by journal and role of journal editors – I see that as a key finding. Detailed comments follow by section. | | | |
| 1. | Abstract Method needs some more specifics eg does not say how identified articles were assessed for inclusion and coded. | We have added in the number of articles analysed, and we have noted the ways in which we identified food industry involvement. | **Lines 31-34**:  All original research articles published in 2018 in the top 10 most-cited nutrition- and dietetics-related journals were analysed. We evaluated the proportion of articles that disclosed involvement from the food industry, including through author affiliations, funding sources, declarations of interest or other acknowledgments. |
| 2. | I would consider including % by journal because I think that is one of the most interesting and important findings – ie journals with FI links publish more FI research favourable to the FI | We have now specifically highlighted in two places in the abstract that the findings varied by journal. | **Lines 39-40**:  196/1,461 (13.4%) articles reported food industry involvement, with large variation by journal.  **Lines 43-44**:  Journals with links to the food industry published a higher proportion of papers with food industry involvement. |
| 3. | Methods I think it would help to know each journal’s position on COI – are authors required to fully disclose COI? Do these journals have any policy on taking food industry-funded/linked research? | We have now assessed the requirements of each journal for authors to disclosure conflicts of interest, and did not ascertain important differences in disclosure requirements.  We also explored whether the journals had specific policies on taking food industry-funded/linked research, but could not find any relevant policies.  In addition, we examined the conflicts of interest disclosures of the editorial board of each journal.  We have added details to the Methods and Results sections explaining the additional analyses we did regarding the policies and disclosures of each journal. | **Lines 119-123**:  We also examined the disclosed conflicts of interest of the editorial board of each of the selected journals, links of the selected journals and their editors to the food industry (based on biographical information provided on the journal website and/or on the website of each editor’s primary affiliation), as well as each journal’s requirements for authors to disclosure conflicts of interest and any other related policies.  **Lines 234-241**:  Each journal had similar policies in place that required authors to disclose conflicts of interest. Four journals (*Advances in Nutrition*, *The Journal of Nutrition*, *Obesity*, *Paediatric Obesity*) included statements regarding conflicts of interest of their editorial board on the journal website. Editors from six journals (*The American Journal of Clinical Nutrition*, *Advances in Nutrition*, *International Journal of Obesity*, *Nutrition Reviews*, *The Journal of Nutrition*, *Obesity*) were identified as having involvement with the food industry (see Table S3). No other relevant policies regarding studies with food industry involvement were identified by any journal. |
| 4. | “food industry actors”: can you include the no. of article assessed (p 5) | We have now included the total number of articles published in the included journals. | **Line 115**:  Details of all articles (n=1,732) published in the selected journals in 2018 were extracted from Medline, CINAHL, Global Health or PubMed. |
| 5. | Does the definition of food industry used have a source you can cite?– is it an accepted definition? | We have now refined our description of our definition of the food industry, and added references. | **Lines 126-134**:  For the purposes of this study, the “food industry” was broadly defined to include all organisations involved in food and non-alcoholic beverage production, distribution, marketing and retail, as well as relevant industry groups and trade associations (28). We included manufacturers of dietary supplements and breast-milk substitutes in this definition. In recognition of the known industry tactic of establishing ‘front groups’ (defined as an organisation that purports to represent one agenda while in reality it serves some other party or interest whose sponsorship is hidden or rarely mentioned) (29), our definition of “food industry” also included organisations that received the majority of their funding from the food industry. |
| 6. | You then include “organisations affiliated with food industry organisations” - I think some clarity around definition here is needed: 1st a definition for “food industry organisations” is needed (this may simply be “ food industry” as you have just defined? Otherwise you need a new definition of “FI organisations”. | We have now refined our description of our definition of the food industry – please refer to response to previous comment. |  |
| 7. | 2nd, I think you also need to define “affiliated” and clarify how you determine which additional organisations are ‘afffiliated’. Ie do you mean “funded by”? And did you need to investigate this? In my experience the funding to some of these organisations is hidden and there is a need to investigate financial links. Some tobacco industry research has developed systems for identifying these financial links which you could perhaps draw on. | We have now provided more detail about how we identified which organisations were part of the food industry. This included use of a pre-existing list of known food industry front groups, as well as online searches of organisational websites.  We have also now noted in the Discussion section that we may not have identified all relevant food industry actors, particularly because it is often difficult to determine financial links. | **Lines 135-143**:  Food industry involvement was determined based on examination of author affiliations, declared funding sources, declarations of interests, and acknowledgements within each article. All organisations identified through these sections of each article were assessed to determine whether they could be classified as part of the food industry. All universities were considered as not part of the food industry. Organisations known by the authors to be part of the food industry as well as those on an established list of known food industry front groups were classified as such (30). Searches of the primary websites of all other organisations were conducted to determine the nature of their operations and their funding sources, where relevant, in order to determine if they could be considered as part of the food industry (31).  **Lines 371-376**:  In addition, our identification of food industry organisations involved in the included studies may have been incomplete. While we made use of an established list of food industry front groups as well as online searches of identified organisations to determine the nature of their operations and funding sources, it has previously been noted that financial links to the food industry are often not publicly available (30). |
| 8. | Food industry involvement: You detail who did much of the coding – eg “two of the authors (DR and GS) to determine whether there was food industry involvement in the paper” and “The categorisation was performed independently by two of the authors (DR and SD), with any discrepancies discussed and resolved with a third author (GS).” But not who coded/double coded whether the findings were favourable. | We have now noted we did the coding of findings in the Methods section. | **Lines 192-193**:  Assessments of principal findings were conducted independently by two of the authors (DR and SD), with any discrepancies discussed and resolved with a third author (GS). |
| 9. | You define a lot of coding categories in the text. It might be worth considering whether this would all be clearer in a table? | Thank you for this suggestion. We have now included Table 1, which provides definitions used to classify principal findings of articles examined, with examples. This replaces text previously contained in the Methods section. | **Line 182:**  **‘Table 1.** Definitions used to classify principal findings of articles examined’ has been added to the manuscript |
| 10. | “The same number of articles with and without industry involvement in each journal was selected for analysis.” – was this overall or by journal? And, I assume this selection effectively came from the pile you had rejected in the 1st run through? Pls can you detail this and give numbers (fine if these go in results). Eg if 1450 articles published and assessed, 196 were food industry linked. The rest then you could select from as non-industry linked? | We have specified in the methods (Lines 183-185) that:  “For each of the ten journals, a sample of randomly selected original research articles that did not report food industry involvement was also selected. An equal number of articles with and without industry involvement in each journal was selected for analysis.”  We have also now added a study flow diagram to the results section that helps make this clearer. | **Line 218:**  ‘Figure 1. Study flow diagram indicating the number of articles included in the study’ has been added to the manuscript |
| 11a | I’m wondering if there are a few minor things you could do to strengthen the paper: In my experience authors might identify themselves as working for a corporation, or funded by a corporation but then declare no COI even when the paper focuses on a topic that is directly in that corporations area of interest (ie where there is in fact a COI). Did you record whether declarations of COIs were consistent with your assessment of whether there was a COI? If not, could you add this? | The primary aim of this study was to identify the extent of food industry involvement in the sample of articles. We identified “involvement” by examining author affiliations, declared funding sources, declarations of interests, and acknowledgements within each article. As we did not set out to examine the veracity of author COI declarations (in the author declarations statements), we did not code the data in such a way that would allow comparison between whether author declarations of conflict of interest were consistent with our assessment of food industry involvement. For example, if food industry funding was noted in an article, we did not code the section of the paper where that declaration was made. Accordingly, we are not able to conduct the suggested analysis without substantial rework. We have noted this as a limitation of the study and identified the value of future work in this area. | **Lines 366-368**:  We did not conduct an analysis of the veracity of each journal’s conflict of interest disclosure requirements, but this warrants further exploration. |
| 11b | I’m interested in the varying percentages of industry linked papers by journal and am glad you present that. Just wondering if there is any evidence of certain editors having links to the FI and whether you would consider exploring and adding that? - Some of the percentages are very high. (I wrote this and then got to the discussion where you mention this! I wonder if it would be more powerful to more formally assess this and include it in the methods and results. Eg you could have a table with journals, % FI linked papers, the % of editors with FI links, other journal links to FI etc.) | Thank you for this suggestion. We have now included an analysis of each journal’s declared links to the food industry and any related policies. Please also refer to our response to your Comment 3.  Note that we did not report the ‘% of editors with links to the food industry’ as this percentage proved difficult to operationalise as the journals have different structures for their editorial boards (e.g. some have associate editors and academic editors etc). Given that it was not a primary focus of our study, we instead identified whether any editors had declared involvement with the food industry and noted other journal links to the food industry (see Table S3). We have noted in the Discussion that the role of editors and their links to the food industry need to be further explored. | Refer to response to Reviewer 1, Comment 3.  Added Table S3: The top 10 most-cited nutrition- and dietetics-related journals in 2018 and their declared involvement with the food industry  **Lines 377-380**:  Finally, we did not conduct a detailed examination of the extent to which the editors of each journal have links to the food industry. Future research should further explore links between journal editors and the food industry and the role of journal editors in assessing conflicts of interest with the food industry. |
| 12. | Results Can you clarify if 1450 is the total no. of articles once editorials, letters excluded? | We have now provided additional details of the number of articles included, including a study flow diagram that illustrates the number of articles analysed. | **Lines 208-209**:  Of the 1,732 articles published in the selected journals, 1,461 peer-reviewed research articles met our inclusion criteria (n=271 excluded) (**Figure 1**)  ‘Figure 1. Study flow diagram indicating the number of articles included in the study’ has been added to the manuscript |
| 13. | Linked to my query about on transparency, you seem to have taken all statements of funding and COI at face value – is that correct? Or did you investigate whether, for example, unknown funders were linked to industry? | Our approach to identifying food industry involvement was through examination of author affiliations, declared funding sources, declarations of interests, and acknowledgements within each article. All organisations that were identified through these data sources were assessed to determine whether they could be classified as part of the food industry. We have provided further details of our methods in this area to be clearer on what we did, and we have noted limitations of our approach in the Discussion section – refer to our response to your Comment 7.  We have noted in the Discussion section (Lines 403-409) that our analyses relied primarily on the self-disclosure of food industry involvement (through declared conflicts of interests, funding acknowledgments, and author affiliations), and undisclosed food industry involvement cannot be captured using the approach we adopted. | Refer to response to Reviewer 1, Comment 7. |
| 14. | Table 1 – can you give %ages in the bottom (totals) line | We have added percentages in the total line for Table 2. | **Table 2**: Percentages added in the total line |
| 15. | Table 3 – ditto – pls give the N total and not just % in the bottom (totals) line | Table 3 has been moved to the Online Supplementary Material as Table S6. We have added “n” in the total line for Table S6. We also added a revised Table 4. | **Table S6:** ‘Nature of the findings in articles with and without 1 food industry involvement, by journal’ -n (total) added in the total line |
| 16. | “Of the 196 randomly selected articles with no industry declared food industry involvement,” 138 (70.4%) had findings considered not applicable to the food industry, 24 (12.2%) reported findings classified as favourable to food industry interests, and 13 (6.6%) reported findings categorised as unfavourable to food industry interests (Table 3).” – I think it is hard to see where these data are in Table 3. Pls consider changing the structure of and/or headings in Table 3 to make this clearer. | We have revised the relevant sections of the results to make the data easier to interpret. This included adding in a new Table 4 and moving the previous Table 3 to the Online Supplementary Material (Table S6). | New **Table 4.** Nature of the findings in articles with and without ^1^ food industry involvement, by type of food industry involvement  **Lines 263-282**:  The majority of papers with food industry involvement reported findings that were considered favourable to the food industry (n=109, 55.6%) (Table 4). The proportion of articles with findings considered favourable to the food industry was even higher (66.2%) where study authors reported either affiliations related to the food industry or direct funding for the study from the food industry (Table 4). In contrast, of the 196 randomly selected articles with no identified food industry involvement, 24 (12.2%, 95% CI 8.0-17.7) reported findings classified as favourable to the food industry.  Only a small proportion (n=13, 6.6%) of papers with food industry involvement reported results that were unfavourable to the food industry (Table 4). The percentage of articles with findings unfavourable to the food industry, mixed findings or neutral findings were similar for those articles with and without food industry involvement (Table 4). 138 (70.4%, 95% CI = 63.5-76.7) articles with no food industry involvement had findings considered not applicable to the food industry, compared to 50 (25.5%) of the articles with food industry involvement. Similar patterns were observed across each journal (Table S6). |
| 18. | Discussion “It has been well documented that a range of industries, including the food industry, seek involvement in research and make use of scientific evidence as part of broader efforts to influence public health policy [16, 26-28].” – I think there are some other important papers to include there. In particular Ulucanlar’s Policy Dystopia Model which identifies “information management” as a key industry tactic – this includes producing & disseminating favourable research. You could also flag that diverse major corporations (including FI) have pushed for policy making systems that provide a route for feeding corporate evidence into policy making <https://journals.plos.org/plosmedicine/article/comments?id=10.1371/journal.pmed.1000202> and indeed have gone on to use those systems (eg <https://journals.plos.org/plosmedicine/article/comments?id=10.1371/journal.pmed.1000202>). Ie the development of favourable evidence is part of a broader system of policy influence. | Thank you, we have added references to those papers. | **Lines 333-337**:  It has been well documented that a range of industries, including the food industry, seek involvement in research, develop evidence that is favourable to their interests, and make use of scientific evidence as part of broader efforts to influence public health policy (19, 22, 29, 41-43). Moreover, there is evidence that major corporations have pushed for policy making systems that provide a route for feeding corporate evidence into policy making (43, 44). |
| 19. | In terms of policy suggestions there is work by Joanna Cohen which explores models through with corporate (in this case tobacco) money can be used to fund research while avoiding COI. <https://tobaccocontrol.bmj.com/content/18/3/228?ijkey=141181f5dd20d7990a3e6b5dbefa6957116251eb&keytype2=tf_ipsecsha>. There are examples of these systems in California and Thailand (mentioned here <https://www.bmj.com/content/353/bmj.i2161>) | Thank you for these suggestions. We have added references to these studies and examples in the manuscript. | **Lines 387-392**:  One option could be to limit industry funding of research to a government- or independently-controlled pool of money that supports a research agenda developed independent of industry, with strict processes to ensure freedom from industry influence (48). A similar model for pharmaceutical research already operates in Italy (49), and in relation to the tobacco and alcohol industry in California and Thailand (50). |
| 20. | In terms of journal recommendations – if you explored whether COIs are actually declared when they exist you could make a recommendation of making clear COI statements. Surely you also need to recommend editors should not have FI links? | Thank you for this suggestion. We have added recommendations for journals on disclosing and managing conflict of interest of editors.  We have also added more detail to the part of the ‘Study limitations’ section that notes that we did not conduct an analysis of the veracity of each journal’s conflict of interest disclosure requirements. | **Lines 366-368**:  We did not conduct an analysis of the veracity of each journal’s conflict of interest disclosure requirements, but this warrants further exploration  **Lines 407-410**:  Journals should also have clear policies on disclosing editorial conflicts of interest, including any links between editors and the food industry. Moreover, any such conflicts need to be actively managed or eliminated. |
| **Reviewer 2** | | | |
| 1. | Bias in nutrition science is an important topic and an under-explored area of research. This article makes a contribution by examining food industry sponsorship/ author conflicts of interest in the top 10 nutrition/dietetics journals. | | |
| 2. | My first reaction to the abstract and introduction is that the authors have overstated the scope of the manuscript. For example, the authors state that "This study aimed to identify the extent of food industry involvement in peer-reviewed nutrition articles." Food industry involvement seems to be operationalized as the relationship between article sponsorship and author conflict of interest compared to favorable/unfavorable "principle findings." This manuscript does not evaluate methodological quality, nor does it evaluate the relationship between sponsorship/COI and study results --therefore it is not analyzing the full extent of food industry involvement. (Furthermore, it may not be possible to know the full extent of industry involvement without access to internal documents). This manuscript would be improved by clearly stating what is analyzed and how it contributes to the overall literature. | We have noted that in the Abstract and the Introduction that the key gap that this research fills is that the extent of food industry involvement in nutrition research has not previously been systematically explored. We have also added more details to the Conclusion section of the Abstract to articulate the implications of this study.  In addition, we have refined the paragraph in the Introduction that articulates the research gap. | **Lines 46-51**:  Food industry involvement in peer-reviewed research in leading nutrition-related journals is commonplace. This study, in line with previous literature, has shown that the results of peer-reviewed studies involving the food industry are more likely to favour relevant food industry interests than peer-reviewed studies without food industry involvement. Given the potential competing interests of the food industry, it is important to explore mechanisms that can safeguard the integrity and public relevance of nutrition research.  **Lines 93-96**:  To date, no study has comprehensively examined the extent and nature of food industry involvement in peer-reviewed research. Better information in this area would assist efforts to assess and manage the potential impact and implications of food industry involvement in research. |
| 3. | Introduction: Line 63 - -It is not clear that the authors are aware of food industry involvement in influencing guidelines for evaluating integrity in nutrition science. For example, citation #8 here is partly sponsored by ASN and ILSI. | We have removed reference to this paper:  Rowe S, Alexander N, Clydesdale F, Applebaum R, Atkinson S, Black R, Dwyer J, Hentges E, Higley N, Lefevre M. Funding food science and nutrition research: financial conflicts and scientific integrity. Nutrition Reviews. 2009;67(5):264-72 | Citation removed |
| 4. | Paragraph at Line 67 -- the list of objectionable motives don't seem very objectionable. Why shouldn't a business entity conduct marketing research? What's wrong with sponsoring research that benefits industry interests? | We have adjusted the wording of that paragraph to make it clearer why business motives may be of concern from a public health perspective. | **Lines 76-86**:  However, from a public health perspective, several concerns have been identified regarding food industry involvement in research. These include: 1) the creation of increased marketing opportunities for industry products, many of which are harmful to population health (15); 2) the establishment and nurturing of relationships between the food industry and nutrition researchers that serves to increase perceived industry credibility, reduce industry criticism, and encourage increased dependency on the food industry (16, 17); 3) industry influence over research agendas to preferentially focus on topics likely to benefit industry interests, rather than topics of public health importance (18); 4) industry influence on the methods, conclusions and impact of research in ways that are likely to favour industry interests over and above other factors (9, 19-21); and 5) use of research for political purposes (22, 23). |
| 5. | Line 86: "To date, no study has comprehensively examined the extent, nature and impact of food industry involvement in nutrition research." --Again, this is an overstatement. No one study could possibly do this. | We have refined this paragraph to better articulate the research gap that this study seeks to address. | **Lines 93-96**:  To date, no study has comprehensively examined the extent and nature of food industry involvement in peer-reviewed research. Better information in this area would assist efforts to assess and manage the potential impact and implications of food industry involvement in research. |
| 6. | Line 88: "This study aimed to fill this significant evidence gap by systematically identifying the extent of food industry involvement in peer-reviewed research articles from a large sample of leading nutrition-related journals." Again -- no one study could fill this gap. This manuscript contributes to a growing body of empirical analysis. It is also important to acknowledge that much "nutrition research" takes place outside of nutrition/dietetics journals. | We have adjusted the description of the aim of the study in line with your suggestion.  We have also noted in the Discussion section that much nutrition research, and much research with food industry involvement, takes place outside of nutrition/dietetics journals. We have also identified several other limitations of the study, in line with your suggestion. | **Lines 97-100**:  This study aimed to contribute to a growing body of empirical evidence related to food industry involvement in peer-reviewed published research by systematically identifying the extent of food industry involvement in research articles from a large sample of leading nutrition-related journals.  **Lines 350-355**:  Importantly, much peer-reviewed nutrition research is published outside of the selected nutrition and dietetics journals. Moreover, the study was not designed to identify research with food industry involvement that is published in topic areas outside of nutrition and dietetics, outside of peer-reviewed journals, or that is funded or conducted by the industry but remains unpublished. Accordingly, the study represents only a small and selected analysis of the extent of food industry involvement in nutrition research.  **Lines 361-363**:  We did not perform any analysis of the appropriateness and rigour of the research methods used in each article. Accordingly, we did not assess the influence of food industry involvement on scientific methods or the way in which they were applied. |
| 7. | Sample: The current SJR list has Annual Review of Nutrition as #1 and Pediatric Obesity at #14 – recommend clarifying at what point in time list was accessed. | We have now noted in the text that we used the journal rankings identified in June 2019. This reflected the rankings applicable to 2018 (the year in which the included articles were published). | **Lines 106-107**:  The study examined articles published in 2018 in the top 10 nutrition and dietetics journals as defined by the SCImago Journal ranking (SJR) as at June 2019. |
| 8. | Line 144: the use of "principal findings" is a bit confusing and does not conform with past evaluations (to my knowledge). If the authors are using an established methodology -- should be referenced. Otherwise -- it is unclear to talk about results contained within conclusions. Results are results. Conclusions are conclusions. See Citation #5 | We have adjusted the wording of the relevant section to be clearer on what we did. Our methods are in line with methods previously used. | **Lines 174-179**:  The principal findings were operationalised as the results that were reported in the ‘results’ section of the abstract of the paper. If the relevant section of the abstract contained insufficient information to deduce the nature of the principal findings, the ‘results’ and ‘discussion’ sections of the paper were also examined to understand the nature of the principal reported findings. This approach was based on methods previously used for similar types of analyses (8, 33). |
| 9. | Results: Line 183: Results would be improved with inclusion of a study flow diagram. Did the authors really assess 1450 articles, or were they screened for food industry involvement and article type? A flow diagram would make it easier to understand the random sample of non-industry involved articles which right now reads like an add on requested by a previous reviewer. The results would also benefit from aligning the flow of tables to match flow of text. Results/tables would also be easier to follow if both n/% are included in Total rows. | As suggested, we have now included a study flow diagram. We have also added more details to the Results section to explain the number of articles assessed.  We have now added more detail to the total rows in Tables 2 and Table S6. We have also revised the text of the Results section to improve clarity. | **Lines 208-209:**  Of the 1,732 articles published in the selected journals, 1,461 peer-reviewed research articles met our inclusion criteria (n=271 excluded) (**Figure 1**).  **Figure 1.** ‘Study flow diagram indicating the number of articles included in the study’ has been added to the manuscript  Table 2 - percentages added to total row.  Results section – various updates for clarity. |
| 10. | Discussion: Line 214 -- would be more accurate to specify that articles without food industry involvement = a random sample. | We have updated the relevant sentence, as suggested. | **Lines 290-297**:  The proportion of articles with findings considered favourable to the food industry was substantially higher among those articles with food industry involvement (55.6%) compared to a random sample of those without (12.2%),%), with the difference even more marked where industry involvement in studies was more direct (author affiliations or direct funding for the study). The percentage of articles considered unfavourable to the interests of the food industry was similar (6.6%) among the articles with food industry involvement and the random sample of those articles without. |
| 11. | Line 226--"importantly, however" -- it is not clear what distinction is being made here. | We have adjusted the wording of the sentence to make the meaning clearer. | **Lines 306-309**:  Other journals included in the sample (*The American Journal of Clinical Nutrition* and *Advances in Nutrition*) are also published by ASN, and had lower proportions of articles with food industry involvement compared to *The Journal of Nutrition*. |
| 12. | Line 236 -- "It has previously been reported that nutrition research funded by the food industry typically respects scientific standards for conducting and reporting scientific studies [14]" This is an industry sponsored study. It should not be taken at face value. | We have made amendments to the relevant paragraph to provide a clearer summary of the literature and its limitations. | **Lines 316-331:**  The findings in this study support existing evidence that research with food industry involvement is generally favourable to the interests of the food industry (8, 11, 18, 21, 24, 26, 38-40). In particular, this study adds to the growing empirical evidence that food industry involvement in nutrition research likely influences research agendas to focus disproportionately on topics of importance to the industry, potentially at the expense of topics of greater public health importance (8, 18). A recent scoping review by Fabbri and colleagues (18) demonstrated the impact of industry involvement across a range of diverse sectors (including medicine and nutrition), finding that industry-funded research was more often focused on products, processes or activities that can be commercialised and marketed, rather than non-market based activities. They concluded that “corporate interests can drive research agendas away from questions that are the most relevant for public health” (18). In addition, food industry-funded research has been noted as often focusing on a specific nutrient, potentially enabling the funder to market the benefits of particular nutrients (24). While it has previously been reported that nutrition research funded by the food industry typically respects scientific standards for conducting and reporting scientific studies (17), the food industry was itself involved in that assessment, and the issue warrants further detailed exploration. |
| 13. | Line 276 - is this a limitation or a continuation of the discussion? | We have added an additional heading and other minor adjustments to the Discussion section to make the flow of text clearer. | **Line 382-384**:  **Implications of the findings**  The finding that food industry involvement is commonplace in peer-reviewed research in leading nutrition-related journals has several implications. |
