# Supplementary material for: The characteristics and extent of food industry involvement in peer-reviewed research articles from 10 leading nutrition-related journals in 2018
Source: PLoS One. 2020 Dec 16;15(12):e0243144. doi: 10.1371/journal.pone.0243144 (PMC7743938; doi:10.1371/journal.pone.0243144)
Supplement: Attachment — Submitted filename: PLOS ONE Round 2 - response to reviewers 27Oct.docx [file pone.0243144.s009.docx]

| **Reviewer comment** | | **Authors’ response** | **Changes made to text** |
| --- | --- | --- | --- |
| **Academic editor** | | | |
| 1. | While one reviewer confirmed that their previous comments had been addressed, unfortunately, not all of the original reviewers were available to re-review the manuscript. Thus, I invited additional reviewers to review the re-submission and ask that you attend to their comments. Particularly, I ask that you re-consider and/or justify the approach to classify a "favourable" conclusion among non-industry-sponsored studies. | See Reviewer 3, Comment 1 – additional details provided, including an additional table. |  |
| 2. | Second, two reviewers have raised concerns about the adequacy of the sampling methods and their reporting and whether these constitute a 'systematic' assessment of the literature; at minimum, please address this in the study's limitations, or, consider revising these methods. | See Reviewer 3, Comment 2 (random sampling) – more detail provided, and Reviewer 4, Comment 8 – addressed in study limitations. |  |
| 3. | The third reviewer raised the question of conducting a regression analysis to infer a causal relationship, which I suggest is beyond the scope of this design, however, I would appreciate your response. | See Reviewer 4, Comment 10 – we agree that this is beyond the scope of the analysis. |  |

| **Reviewer 3** | | | |
| --- | --- | --- | --- |
| In an important contribution to understanding of the role of commercial actors in science, the authors map the extent and nature of food industry involvement in the area of nutrition research. They identify peer-reviewed articles which declare food industry funding or links, in 10 leading nutrition journals, and categorise whether their findings are favourable the food industry. They compare the latter to a sample of articles which do not declare industry involvement, concluding that articles with industry involvement are more likely to be favourable to the food industry than those without. They also find that the extent of food industry involvement nutrition-related research is substantial, though it varies between journals.  Overall, the paper is well written, a pleasure to read, and fills a crucial research gap. The rationale is clear and, for the most part, the authors explain very well what they did. The classification of food industry involvement is very clear and detailed, which provides interesting insights into which parts of the industry are most involved in nutrition research and will be useful for other researchers looking to classify food industry actors. | | | |
| 1. | The key point I would like to raise pertains to the analysis of the articles without food industry involvement for favourability. The definition used for the industry-funded/linked articles seems appropriate as the favourability of their findings can be assessed in relation to the associated food industry actor. Favourability for the articles without industry involvement, however, is defined as follows: “a favourable finding for any food product or nutrient was considered favourable to the food industry, whereas a negative finding for any food product or nutrient was considered unfavourable” (line 188 onwards). This approach to operationalising favourability to the food industry is very broad and raises the question whether articles supporting the consumption of nutrients and products widely considered as healthy, such as whole grains, fruits, and vegetables, would be included as favourable to the food industry. I suggest that the authors refine this part of the paper accordingly. This could be done, for example, by clarifying or revisiting the definition of favourability for articles without food industry involvement, or by expanding the reporting of results on favourability for articles without industry involvement (i.e. which products/nutrients did the findings support). | For the purposes of assessing the extent to which articles without food industry involvement favoured food industry interests, we deliberately adopted a broad interpretation of food industry interests. In response to the reviewer’s comments, we have provided more detail in the methods regarding our approach. We have also now included an additional table (Table S6) which provides more detailed results of the analysis of articles without food involvement, including a breakdown of the particular foods, food components and nutrients that were the topic of relevant papers. | **Methods, Lines 200-208**:  The principal findings of all selected articles without food industry involvement were examined and classified in the same way as the principal findings of the articles with food industry involvement. As there was no specific industry actor involved in these articles, a broad interpretation of food industry interests was taken when assessing the extent to which articles favoured food industry interests. For example, a favourable finding for any food product or nutrient was considered favourable to the food industry, whereas a negative finding for any food product or nutrient was considered unfavourable. The primary topic area of each of the articles was noted, including the particular foods, food components or nutrients (as relevant).  **Results, Lines 285-290**:  In contrast, of the 196 randomly selected articles with no identified food industry involvement, 19 (9.7%, 95% CI: 7.0-12.4) reported findings classified as favourable to the food industry. The vast majority (n=15/19, 78.9%) of these articles related to particular nutrients and/or food components (e.g., protein, vitamins), with the remaining four articles (21.1%) relating to foods and food products (e.g., coffee, green tea) (**Table S6**).  **Table S6 added** |
| 2. | Additionally, it would be helpful to have more detail regarding the process behind the selection of the random sample of articles without food industry involvement (line 183) | We have now added in more detail of the process behind the selection of the random sample of articles without food industry involvement. | **Methods, Lines 194-200**:  For each of the ten journals, a sample of randomly selected original research articles that did not report food industry involvement was also selected. The process for selection of these articles was that, first, the number of articles with food industry involvement for each journal was calculated. Then, the matching number of articles from each journal, but without food industry involvement, was selected randomly from the list of included articles using the RAND function in Excel. Accordingly, an equal number of articles with and without industry involvement in each journal was selected for analysis. |
| **Reviewer 4** | | | |
| 1. | Sacks and colleagues have conducted an important piece of work to describe characteristics, such as authors' affiliations, funding source from food industry, and research findings regarding the food intervention/exposure, in academic articles published in the top 10 nutrition journals. While it is important to describe these characteristics, it is also important to understand whether there is any causal inference can be established in this setting. | | |
| 2. | Abstract The study has used nature and extent in the objective of the study frequently. However, this is not clear to the readers what the authors are trying to measure. Suggest using more precise wording to describe what the study measured. | We have adjusted the title of the study to be more specific. Please also refer to Reviewer 4, Comment 6. | Title amended to:  The characteristics and extent of food industry involvement in peer-reviewed research articles from 10 leading nutrition-related journals in 2018 |
| 3. | Abstract  Methods: if a systematic search was applied to identify the articles, please state it here. Otherwise, please describe how the articles were selected and what are the principal search articles and a random sample of studies. | We included all original research articles published in the selected journals (top 10 most-cited nutrition- and dietetics-related journals) in 2018. | No changes made to text |
| 4. | Abstract  Results: some of the values should be provided. For example, please provide values for the variation related to “196/1,461 (13.4%) articles reported food industry involvement, with large variation by journal.” and values of proportion for “Journals with declared links to the food industry published a higher proportion of papers with food industry involvement. | We have revised the abstract to add more details of the variation by journal. In order to comply with the word limit (300 words) we were not able to add further details. | **Abstract, Lines 41-44**:  The extent of food industry involvement varied by journal, with *The Journal of Nutrition* (28.3%) having the highest and *Paediatric Obesity* (3.8%) having the lowest proportion of industry involvement. |
| 5. | Abstract  Conclusion: it needs more substantive evidence, such as regression analysis, to substantiate the conclusion: “This study, in line with previous literature, has shown that the results of peer-reviewed studies involving the food industry are more likely to favour relevant food industry interests than peer-reviewed studies without food industry involvement.” | We have amended the text to ensure it reflects the results of this study more precisely. | **Abstract, Lines 51-54**:  In line with previous literature, this study has shown that a greater proportion of peer-reviewed studies involving the food industry have results that favour relevant food industry interests than peer-reviewed studies without food industry involvement. |
| 6. | Introduction: line 93, page 11: Again, I find it hard to understand what it means or what is measured here by using "extent" and "nature" in the objective of the study. | The extent of food industry involvement refers to the proportion of peer-reviewed research with food industry involvement, while the nature of that involvement refers to the characteristics of food industry actors that are involved in research and the different ways in which they are involved (e.g. study authorship, different types of funding provided).  In response to the reviewer’s feedback, we have edited the relevant section of the text to provide greater clarity. | **Introduction, Lines 98-104**:  To date, no study has comprehensively examined the extent and nature of food industry involvement in peer-reviewed research. Better information regarding the extent of food industry involvement, characteristics (e.g. industry sector, company size) of food industry actors that are involved in nutrition-related research, and the ways in which they are involved (e.g. study authorship, different types of funding provided) would assist efforts to assess and manage the potential impact and implications of food industry involvement in research. |
| 7. | Line 95: “Better information in this area” – please be specific about what “better” information means here. This will also help the readers understand the research gap in the literature. | Please refer to our response to the previous comment. |  |
| 8. | Methods: Please provide rationale of only looking into 10 nutrition journals rather than conducting a systematic review to identify nutrition studies. I would also recommend describing the study design and intervention/exposure of the included studies. | The study was exploratory in nature, and is the first study to systematically examine the extent of involvement of the food industry in peer-reviewed research articles published in the leading nutrition and dietetics journals. We have acknowledged in the Discussion section (study limitations) that much peer-reviewed nutrition research is published outside of the selected nutrition and dietetics journals. In response to the reviewer’s feedback, we have added a comment that future studies should investigate articles from both nutrition and non-nutrition journals (including, for example, journals in Medicine and Public Health).  While we agree that it would be interesting to describe the study design and intervention /exposure of the included studies, we did not extract that information. In response to the reviewer’s feedback, we have added a comment that future studies should collect this information and conduct analysis by study design type. | **Discussion, Lines 379-383**:  Future studies should investigate nutrition-related articles from journals with both a nutrition and non-nutrition focus (including, for example, journals in medicine and public health). Ways to automate methods for comprehensively identifying different types of food industry involvement in published studies need to be explored.  **Discussion, Lines 389-394:**  We did not perform any analysis by study design of the included articles or in relation to the appropriateness and rigour of the research methods used in each article. Accordingly, we did not assess the influence of food industry involvement on scientific methods or the way in which they were applied. Aspects of study design and specific mechanisms by which food industry involvement may influence study focus areas and results should be included in future studies. |
| 9. | Details are needed to describe how the information was obtained in line 119-123. | We have now added in details of how the information regarding each journal and their editorial boards was obtained. | **Methods, Lines 127-133**:  We also examined the disclosed conflicts of interest of the editorial board of each of the selected journals (based on information provided on the website of each journal), links of the selected journals and their editors to the food industry (based on biographical information provided on the journal website and/or on the website of each editor’s primary affiliation), as well as each journal’s requirements for authors to disclosure conflicts of interest and any other related policies (based on information provided on the website of each journal). |
| 10. | Since the authors are assessing how industry involvement in nutrition research may affect “favorable” findings, is it possible to conduct a regression analysis to infer a potentially causal relationship? | This paper aimed to identify the extent of food industry involvement in nutrition research, and the extent to which research findings support food industry interests for both articles with declared food industry involvement, and those with no declared food industry involvement. Due to the research methods selected and the cross-sectional nature of the data, the study was not designed to assess a causal relationship. | No changes made to text |
| 11. | Table 2: can the authors provide 95% CI for the articles with food industry involvement, so that it is comparable with the results in Table 4? | Confidence intervals are only applicable when the estimate is from a sample drawn from a larger population. In this case, we identify all articles with declared food industry involvement from the population of articles in the selected journals, and so the results are not estimates but actual values, and we do not believe it would be appropriate to include confidence intervals. We have added a note to Table 4 to explain this. | Table 4, footnote added:  ^2^ 95% confidence intervals are not provided for articles with food industry involvement because we identified all articles with declared food industry involvement from the population of articles in the selected journals. |
| 12. | Table 3: what does “other” include? | Table 3 includes a footnote that food industry sector definitions are provided in Table S1 and a list of identified organisations within each food industry sector are provided in Table S2. | No changes made to text |
| 13. | Discussion I think one of the major limitations of the study is the selection of articles. The current included studies may not be complete, as many nutrition-related research articles are not necessarily published in nutrition journals but in other journals with a broader category in health. These may include high-impact medical journals and science journals. | We acknowledge that much peer-reviewed nutrition research is published outside of the selected nutrition and dietetics journals, and note in the study limitations (Lines 374-379) that the study represents only a small and selected analysis of the extent of food industry involvement in nutrition research. In response to the reviewer’s feedback, we have added a comment that future studies should investigate articles from both nutrition and non-nutrition journals (including, for example, journals in Medicine and Public Health). | **Discussion, Lines 379-383**:  Future studies should investigate nutrition-related articles from journals with both a nutrition and non-nutrition focus (including, for example, journals in medicine and public health). Ways to automate methods for comprehensively identifying different types of food industry involvement in published studies need to be explored. |
